# Traditional Chinese Medicine: A Class of Potentially Reliable Epigenetic Drugs

**DOI:** 10.3389/fphar.2022.907031

**Published:** 2022-06-14

**Authors:** Daoqi Zhu, Aiwu Li, Ying Lv, Qin Fan

**Affiliations:** ^1^ School of Traditional Chinese Medicine, Southern Medical University, Guangzhou, China; ^2^ Nanfang Hospital, Southern Medical University, Guangzhou, China

**Keywords:** traditional Chinese medicine, DNA methylation, epigenetic drugs, anti tumor drug, Chinese herb formula

## Abstract

Epigenetic modification, especially DNA methylation, plays a nonnegligible role in the occurrence and development of tumors. Increasing studies are indicating that traditional Chinese medicine (TCM) plays a considerable anti-tumor role by regulating the process of DNA methylation modification. Studies on TCM regulating DNA methylation modification mostly focus on the whole genome and abnormal methylation status by active ingredients or single compounds and Chinese herb formula (CHF). The balance and overall concept of TCM theory coincides with the balance of DNA methylation modification in the tumor environment. Regardless of how TCM modulates epigenetics in tumor, it has been shown to bet a class of potentially reliable epigenetic drug.

## Introduction

Epigenetics is the study of how the environment or behaviors regulate gene expression without changing the DNA sequence. Common types of epigenetic modification include DNA methylation, histone modification, non-coding RNA regulation, and m6A RNA methylation (Nebbioso et al., 2018). Epigenetic modification, especially DNA methylation, plays a nonnegligible role in the occurrence and development of tumors (Papanicolau-Sengos and Aldape, 2022). Therefore, maintaining or reversing the process of DNA methylation has become a new strategy for tumor therapy. DNA methylation is a biological process by which methyl groups are added to the DNA molecule (most commonly cytosine) by DNA methyltransferases (DNMTs) (Moore et al., 2013). DNA demethylation is the process of removal of a methyl group from cytosines and can be passive or active. Active DNA demethylation modification occurs through oxidation by a family of proteins called ten-eleven translocation methylcytosine dioxygenases (TET). DNA methylation is strictly controlled, but a large number of DNA methylation deletions have been found in the whole genome of tumors. The character of global hypomethylation of DNA in cancer was the initial epigenetic abnormality recognized in human tumors (Nebbioso et al., 2018). The hypomethylation of cell-growth-related genes will lead to cell abnormal proliferation, and lead to the occurrence and development of cancer. Hypermethylation was frequently found in tumor suppressor genes related to DNA repair and cell division control, while also increasing the risk of tumor development.

TCM is widely used in China and many other Asian and Western countries, and the holistic, dynamic, individual, and interaction with the environment characteristics of epigenetics are highly consistent with the core concept of TCM. With increasing studies on the anti-tumor effect of TCM, many reports indicated that TCM plays a considerable anti-tumor role by regulating the process of DNA methylation modification. But how do the different types of TCM work in regulating DNA methylation modification?

## The Effect of Traditional Chinese Medicine on the Regulation of DNA Methylation Modification in Tumors

As the study of human diseases spectrum and life sciences has progressed, scientists gradually recognized single-factor and single-target treatment models were limited for diseases with complex pathological mechanisms, especially tumors. The multi-component and multi-target characteristics of TCM make it difficult to develop drug resistance with fewer side effects, and it shows unique advantages in the treatment of complex diseases, especially tumors (Huo et al., 2022). Studies on TCM regulating DNA methylation modification mostly focus on the whole genome and abnormal methylation status by active ingredients or single compounds and CHF (Xiang et al., 2019). The mechanism of this process is mainly by regulating the expression of DNMTs or the related signaling pathways (Dammann et al., 2017).

Turmeric (Jiang Huang in Chinese) is the rhizomes of *Curcuma longa*, which is commonly used for promoting blood circulation and removing blood stasis in TCM (Prasad and Aggarwal, 2011). The main active ingredient curcumin has been widely studied due to its excellent anti-tumor and antioxidant activity (Yang et al., 2020; Zhu et al., 2020). Curcumin inhibits DNA methylation by covalently blocking the catalytic thiolate of DNMT1 and reducing the expression of tumor suppressor gene Sp1 Transcription Factor (Sp1) and Transcription factor p65 (p65) in acute myeloid leukemia (AML) cell lines *in vitro* and *in vivo* (Yu et al., 2013). Moreover, curcumin shows demethylation effect in colorectal cancer and glioblastoma by inhibiting the expression of DNMTs (Link et al., 2013; Wu et al., 2013). *Angelica sinensis* (Dang Gui in Chinese) is the rhizomes of *Angelica sinensis* (Oliv.) Diels, which is a blood-tonifying and blood-activating drug often used to treat breast cancer in TCM (Yue et al., 2019). Z-Ligustilide extracted from *Angelica sinensis* can inhibit the growth of mouse prostate cancer cells by demethylation of the promoter of the tumor suppressor gene NFE2 Like BZIP Transcription Factor 2 (NrF2) (Su et al., 2013). Ginseng (Ren Shen in Chinese), which is a rhizome from the Araliaceae and has multiple anti-tumor effects (Hong et al., 2021), should be one of the most studied TCMs. Ginsenoside Rg3 was one of main ingredients found to have a global hypomethylation effect in human hepatoma cell line HepG2 (Teng et al., 2017). It has also been proven to directly down-regulate methylation of Von Hippel-Lindau Tumor Suppressor (VHL) or indirectly decrease the expression of HIF-1α through reducing methylation of miR-519a-5p precursor gene by inhibiting the expression of DNMT3A in ovarian cancer cells (Wang et al., 2021). Another ingredient, Ginsenoside Rh2, shows global hypomethylation effects on breast cancer cells MCF-7 by regulating cell-mediated immune pathway (Lee et al., 2018). See Table 1.

**TABLE 1:** DNA methylation regulation by TCM.

TCM	Species, concentration	Quality control reported? (Y/N)	Genes	Target	Methylation modification	Disease or pharmacological effect
curcumin	Turmeric, rhizomes of Curcuma longa	Y-Prepared according to Chinese Pharmacopoeia (2020) pharmacopeia	Sp1, p65, genome-wide, RANK	DNMT1	Inhibit methylation, Methylation, Demethylation	Acute myeloid leukemia (AML), Colorectal Cancer, Glioblastoma
Z-ligustilide	Root of *Angelica sinensis* (Oliv.) Diels, rhizomes of Ligusticum chuanxiong Hort	Y-Prepared according to Chinese Pharmacopoeia (2020) pharmacopeia	Nrf2	N.A.	Inhibit methylation	Prostate cancer
Ginsenoside Rg3, Ginsenoside Rh2	Ginseng, root of *Panax ginseng* C. A. Mey	Y-Prepared according to Chinese Pharmacopoeia (2020) pharmacopeia	genome-wide, VHL, miR-519a-5p	DNMT3A	Hypomethylation	Hepatoma, Ovarian, Breast cancer
Liuwei Dihuang Wan	Prepared rhizome of *Rehmannia glutinosa* (Gdertn) Iibosch., 2.88 g Fruit of Cornus officinalis Sieb. et Zucc., 1.44 g rhizoma of Dioscorea opposita, 1.44 g Bark of Paeonia suffruticosa Andr., 1.08 g Wolfiporia cocos (Schw.) Ryv.&Gibn, 1.08 g Rhizome of Alisma orientale (Sam.) Juz. 1.08 g	Y-Prepared according to Chinese Pharmacopoeia (2020) pharmacopeia	ERα	DNMT1	Inhibit methylation	anti-atherosclerosis
Wu Tou decoction	Herbaceous stems of Ephedra sinica Stapf, 9 g Rhizome of Paeonia Lactiflora, 9 g Rhizome of Astragalus membranaceus (Fisch.), 9 g Rhizome of Glycyrrhiza uralensis Fisch. 9 g Root of RadixAconiti 6 g	Y-Prepared according to Chinese Pharmacopoeia (2020) pharmacopeia	genome-wide	DNMT1	Inhibit methylation	Rheumatoid arthritis, Anti-inflammatory
Baihu Guizhi Decoction	Rhizome of Anemarrhena asphodeloides Bge. 18 g Gypsum fibrosum 30 g Rhizome of Glycyrrhiza uralensis Fisch. 6 g Seed of Oryza sativa subsp. Japonica 6 g Twig of Cinnamomum cassia Presl 9 g	Y-Prepared according to Chinese Pharmacopoeia (2020) pharmacopeia	genome-wide, AHCY, RPL3	N.A	Inhibit methylation	Febrile arthralgia
Shengmai Yin	Root of Panax ginseng C. A. Mey 9 g Root of Ophiopogon japonicus (Thunb.) Ker-Gawl. 9 g Fruit of Schisandra chinensis (Turcz.) Baill. 6 g	Y-Prepared according to Chinese Pharmacopoeia (2020) pharmacopeia	TNC	DNMT3A	methylation	Nasopharyngeal carcinoma

CHF is a characteristic representation of TCM, with increasing evidence showing CHFs are involved in the DNA methylation modification of various diseases. However, how CHF affects DNA methylation modification in tumor still needs to be further clarified. Liuwei Dihuang Wan has the effects of nourishing yin and tonifying kidney in TCM and can be used for anti-atherosclerosis by decreasing methylation of Estrogen receptor alpha (ERα) through inhibiting the expression of DNMT1 (Chen et al., 2020). Wu Tou decoction was a common prescription for cold-dampness and obstruction of joints. Which can treat rheumatoid arthritis because the anti-inflammatory effects regulate DNA methylation and histone modifications (Liu et al., 2016). Baihu Guizhi Decoction (BGD) is a common CHF used for clearing away heat and relieving pain. The study on the characteristic methylation genes of febrile arthralgia (PA) model by BGD showed that the methylation status of CpG islands in the whole genome of the PA group was lower than that of the standard group (Chen et al., 2017). It is indicated that BGD achieves the therapeutic effect on PA by regulating the methylation level of characteristic genes. Since epigenetics characters of tumor is extensive hypomethylation and hypermethylation of a few tumor suppressor genes, most studies were focused on antitumor by regulating demethylation of tumor suppressor genes,rather than methylation of oncogene. As an anti-tumor CHF prescription, Shengmai Yin is a kind of tonic which has the effects of invigorating qi and invigorating the pulse, nourishing yin, and invigorating body fluid in TCM. Tenascin C (TNC) encodes an extracellular matrix protein with a spatially and temporally restricted tissue distribution which play an indispensable role in fetation and rarely express in adult due to hypermethylation (Lind et al., 2013). However, the high expression of TNC in nasopharyngeal carcinoma (NPC) cell line CNE-2 caused by abnormal demethylation induced radioresistance. A previous study (Liu et al., 2021) indicated that Shengmai Yin radiosensitized NPC radioresistance cell line by partial hypermethylation of TNC, which suggests Shengmai Yin can be used as a potential radiosensitizer or epigenetic drug for NPC. The goal of epigenetic therapy is “epigenetic reprograming” by a drug that reverses abnormal epigenetic activity in cancer cells and the critical benefit of epigenetic drugs is their innocuity. See Table 1. TCM be used as a class of potentially reliable epigenetic drug which is consistent with the current trend of tumor treatment - radiotherapy and chemotherapy combined with epigenetic drug therapy.

## Discussion

The theory of TCM believes that yin and yang are in constant motion and flux. In clinical practice, balancing yin and yang to achieve “the harmony of yin and yang” is the ultimate goal of TCM treatment of diseases. The methylation and demethylation of DNA are also in dynamic flux and this characteristic has a natural and vague connection with the theory of TCM. Both target the demethylation of tumor suppressor genes and reverse the hypomethylation state of oncogenes, which are new strategies for epigenetic drug-assisted tumor therapy. TCM has obvious effects and advantages in this process. The basic theory of TCM believes that syndromes are the reflection of a staged pathology in which the internal and external environment and the human body interact, which can adjust the performance of syndromes, thereby affecting the occurrence and development of complex diseases. This view is in line with the idea of reversible regulation by epigenetics. The lasting effect on improving people’s physique through multi-target regulation is based on TCM theory. In addition to the application research of CHF, DNA methylation modification can also provide a theoretical basis for the rationality of modern CHF, such as rational optimization of prescriptions according to the different epigenetic effects and preferences of different main components of drugs. Therefore, we can provide new approaches for the research and development of TCM as epigenetic drugs according to the ideas of epigenetics.

## Conclusion

The balance and overall concept of TCM theory coincides with the balance of DNA methylation modification in the tumor environment. Regardless of how TCM modulates epigenetics in tumor, it has been shown to be a class of potentially reliable epigenetic drug.

## Abbreviations

CHF, chinese herb formula; BGD, baihu guizhi decoction; DNMTs, DNA methyltransferases; TCM, traditional chinese medicine; TET, ten-eleven translocation methylcytosine dioxygenases.
